# Demonstration of *In Vitro* to *In Vivo* Translation of a TYK2 Inhibitor That Shows Cross Species Potency Differences

**DOI:** 10.1038/s41598-020-65762-y

**Published:** 2020-06-02

**Authors:** Brian S. Gerstenberger, Mary Ellen Banker, James D. Clark, Martin E. Dowty, Andrew Fensome, Roger Gifford, Matthew C. Griffor, Martin Hegen, Brett D. Hollingshead, John D. Knafels, Tsung H. Lin, James F. Smith, Felix F. Vajdos

**Affiliations:** 1 Medicine Design, Pfizer Inc, 1 Portland Street, Cambridge, Massachusetts 02139 United States; 2 Medicine Design, Pfizer Inc, Eastern Point Road, Groton, Connecticut 06340 United States; 3 Inflammation and Immunology, Pfizer Inc, 1 Portland Street, Cambridge, Massachusetts 02139 United States; 4 Drug Safety Research and Development, Pfizer Inc., 1 Portland Street, Cambridge, Massachusetts 02139 United States

**Keywords:** Drug discovery, Immunology

## Abstract

Translation of modulation of drug target activity to therapeutic effect is a critical aspect for all drug discovery programs. In this work we describe the profiling of a non-receptor tyrosine-protein kinase (TYK2) inhibitor which shows a functionally relevant potency shift between human and preclinical species (e.g. murine, dog, macaque) in both biochemical and cellular assays. Comparison of the structure and sequence homology of TYK2 between human and preclinical species within the ATP binding site highlights a single amino acid (I960 → V) responsible for the potency shift. Through TYK2 kinase domain mutants and a TYK2 980I knock-in mouse model, we demonstrate that this single amino acid change drives a functionally relevant potency difference that exists between human and all evaluated preclinical species, for a series of TYK2 inhibitors which target the ATP binding site.

## Introduction

Success in a drug discovery project depends upon target validation, an efficient and validated screening funnel, and confidence in translation from preclinical studies to human. Historically, preclinical animal models (protein, cells, and *in vivo* models) have played a central role in both supporting the therapeutic rationale of drug targets and evaluating the efficacy of proposed drug molecules^1^. A significant challenge can arise using preclinical animal models when key residues of the protein sequence of the target differ from human. Understanding the effect of these amino acid differences on binding and activity is pivotal to the successful utilization of murine and other preclinical species within a drug discovery program^2^. During our efforts toward developing an inhibitor of non-receptor tyrosine-protein kinase (TYK2), we discovered a series of compounds that demonstrated reduced potency in several species compared to human. Through sequence alignment analysis, X-ray crystallography and biochemical mutation studies, cross species *in vitro* cellular work, and ultimately *in vivo* studies with a TYK2 knock-in mouse model, we attributed this effect to a single amino acid difference in the ATP binding site of TYK2. This understanding was key to building our confidence in translation to human for this series, and highlighted challenges in interpreting results from preclinical studies for this target^3,4^.

**Figure 1:**
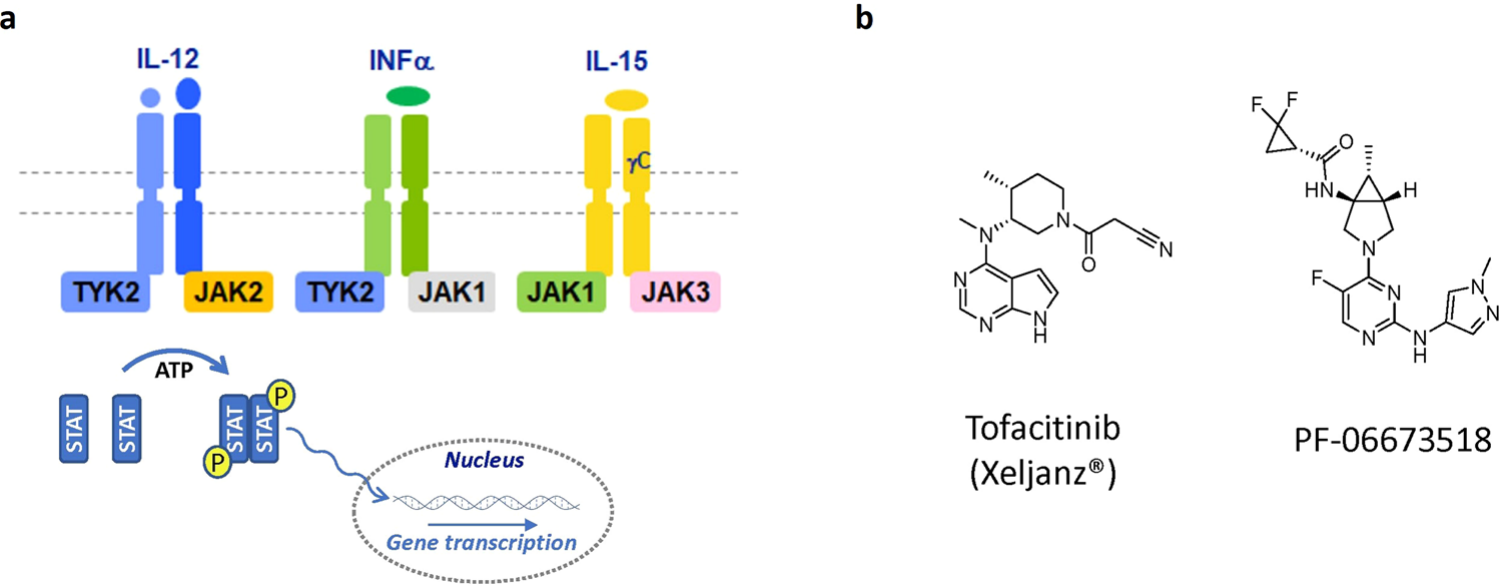
(**a**) Subset of JAK signaling partners in the JAK-STAT signaling pathway; (**b**) Structure of Tofacitinib and PF-06673518.

A number of autoimmune diseases have been linked to or regulated by immune cell responses mediated by intracellular cytokine signaling pathways^5^. The Janus kinase (JAK) family, which includes JAK1, JAK2, JAK3 and TYK2, is an important component of signaling pathways associated with the intracellular domain of the cytokine receptors^6^. Of the four family members, JAK1, JAK2, and TYK2 are ubiquitously expressed whereas JAK3 is confined to hematopoietic, myeloid, and lymphoid cells. Seven regions of sequence similarity have been found between the Janus kinases and designated Janus homology (JH) domains. The carboxy-terminal JH1 domain is a tyrosine kinase domain adjacent to an inactive pseudokinase domain (JH2)^7^. The pseudokinase domain usually negatively regulated the functional protein kinase domain. TYK2 controls the signaling downstream of the receptors for type I interferons (IFNs), interleukin (IL)-12 and IL-23, which are critical in the pathobiology of multiple autoimmune diseases. In these disorders, a key pathogenic role for T helper 1 (Th1) cells and Th17 cells in mediating inflammation and tissue injury has been implicated. IL-12 and IL-23 are critical in the expansion and survival of pathogenic Th1 and Th17 cells, respectively. Additionally, genome-wide association studies indicate that a deactivating TYK2 variant provides protection from several autoimmune diseases^8^. Pairs of JAK kinases bind to the intracellular domains of cytokine receptors and mediate cytokine signaling via phosphorylation and activation of Signal Transducer and Activator of Transcription (STAT) transcription factors (Fig. 1a). TYK2 and JAK1 associate with cytokine receptors for type I IFNs and IL-10. TYK2 can also associate with JAK2 to transduce signals from receptors for IL-12 and IL-23. JAK1 pairs with JAK2 to mediate signaling via receptors for the IL-6 family of cytokines and for IFNγ. JAK3 only pairs with JAK1 to transduce signals through the common γ-chain containing cytokine receptors for IL-2, IL-4, IL-7, IL-9, IL-15 and IL-21. JAK2 homodimers are critical for the signaling of hematopoietic cytokines and hormones including erythropoietin, thrombopoietin, granulocyte-macrophage colony-stimulating factor, prolactin and growth hormone.

Multiple JAK inhibitors such as tofacitinib (XELIJANZ) (**1**), baricitinib (OLUMIANT), ruxilitinib (JAKAFI), upadacitinib (RINVOQ) have been approved for the treatment of inflammatory and myeloproliferative diseases^9^. A selective inhibitor of TYK2 is of clinical interest due to its potential for blocking proinflammatory cytokine signaling from Type I IFN, IL-12 and IL-23^10^. We have developed a series of aminopyrimidinyl inhibitors which bind to the ATP site of TYK2 and JAK1 kinases to block ATP binding^11^. This led to the discovery of a dual TYK2/JAK1 inhibitor PF-06673518 (compound 19) and subsequent clinical candidates (Fig. 1b)^12,13^.

Initial experiments with PF-06673518 showed a significant loss of enzymatic potency in mouse TYK2 (846 nM) when compared to human TYK2 (29 nM), which complicated our interpretation of certain preclinical pharmacology data. To provide a rationale for this potency shift we undertook a thorough analysis of cross-species protein sequence alignment and protein-ligand structure, ultimately generating mutant protein constructs and a mutant mouse strain to validate our hypothesis. The TYK2 knock-in mouse was key to building confidence in the ability of PF-06673518 to inhibit this pathway in an appropriate *in vivo* model^14^.

## Results and Discussion

Identification and discovery of PF-06673518 as a potent TYK2 inhibitors was accomplished through the optimization of an aminopyrimidine series^12^. As the project advanced, efforts turned to investigating the performance of PF-06673518 in *in-vivo* inflammation models. Before undertaking *in vivo* work in mice, the biochemical potency of PF-06673518 in mouse wild type (WT) TYK2 was determined to compare with human TYK2. Previously reported biochemical profile of tofacitinib across the four human JAK kinases are shown in Table 1^15^. Tofacitinib has comparable, moderately potent inhibition of both human TYK2 (IC_50_ = 489 nM) and mouse WT TYK2 (IC_50_ = 966 nM). PF-06673518 is a potent inhibitor of human WT TYK2 (IC_50_ = 29 nM) and JAK1 (IC_50_ = 41 nM). In contrast to tofacitinib however, PF-06673518 is approximately 48-fold less potent against WT mouse TYK2 (IC_50_ = 1,407 nM) than human. This loss of potency for PF-06673518 was unexpected as tofacitinib also binds to the ATP pocket of the Janus Homology 1 (JH1) domain of TYK2.

**Table 1:** Biochemical potency of tofacitinib^15^ and PF-06673518 across wild type human TYK2 and mouse wild type TYK2; Compounds were assayed at least three times, and the IC_50_ reported as the geometric mean along with ± standard deviation.

Compound	Human WT				Mouse WT
	JAK1 IC_50_ (nM)	JAK2 IC_50_ (nM)	JAK3 IC_50_ (nM)	TYK2 IC_50_ (nM)	TYK2 IC_50_ (nM)
Tofacitinib	15	77	55	489	966 ± 157
PF-06673518	41 ± 15	652 ± 175	4,267 ± 1,060	29 ± 11	1,407 ± 472

ATP concentration = 1 mM.

Due to this observed loss in mouse biochemical potency for PF-06673518 we turned our attention to understand possible cellular potency impacts. Since the JAK-STAT pathway functions through a pairing of two JAK kinases, the IC_50_ for IL-12 signaling (TYK2-dependent) and IL-15 signaling (TYK2-independent) were determined to understand the contribution of TYK2 to cellular activity. Using leukocytes isolated from human and mouse the inhibition of IL-15 (JAK1/JAK3) and IL-12 (TYK2/JAK2) induced phosphorylation of STATs was determined for tofacitinib and PF-06673518. To eliminate further complications from protein binding and red blood cell partitioning the cellular assays were performed in the absence of serum or other proteins.

Both tofacitinib and PF-06673518 are potent inhibitors of JAK1 and show inhibition of IL-15-inducted pSTAT5 in human leukocytes (IC_50_ = 32 and 135 nM respectively) (Table 2). The IL-15 IC_50_ values for PF-06673518 are nearly identical for both human and mouse at 135 and 127 nM. In the case of the inhibition of IL-12-induced pSTAT4, which requires TYK2 and JAK2, tofacitinib again shows consistent potency (ie <two-fold difference) across species, here partially attributed to its inhibition of JAK2. In contrast, PF-06673518 drives inhibition of IL-12-induced pSTAT4 primarily via inhibition of TYK2 and demonstrated approximately a 10-fold higher potency in human cells (IC_50_ = 64 nM) than in mouse (IC_50_ = 518 nM). It is noted that the magnitude of the shift in cellular potency is smaller than the observed biochemical shift. This may be attributed to partial contribution of the cellular activity driven *via* JAK2 inhibition in the mouse. Similar loss of potency for the inhibition of IL-12 by PF-06673518 but not in IL-15 were also observed in monkey and dog experiments (see SI Table 2). Closer examination of the binding mode of PF-06673518 in TYK2 in protein X-ray structure and possible species differences were examined to understand these observations.

**Table 2:** Phospho-STAT inhibition in lymphocyte assays - of tofacitinib^15^ and PF-06673518 across wild type human TYK2 and mouse wild type TYK2. Values represent geomean IC_50_ (nM) ± standard deviation from four experiments.

Compound	IL-12 (TYK2/JAK2) induced pSTAT4 IC_50_ (nM)		IL-15 (JAK1/JAK3) induced pSTAT5 IC_50_ (nM)	
	Human WT	Mouse WT	Human WT	Mouse WT
Tofacitinib	145 ± 34	257 ± 34	32.1 ± 4.4	39.5 ± 6.7
PF-06673518	64.3 ± 10.0	518 ± 84	135 ± 15	127 ± 6.7

### Structural alignment

**Figure 2:**
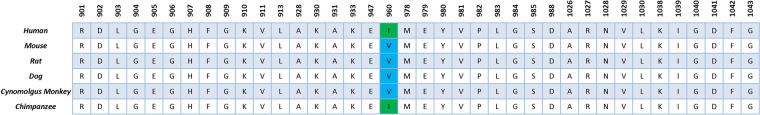
Sequence Alignment of TYK2 ATP binding site across spices (TYK2 human AA numbering)^16^.

We hypothesized that the loss in potency for mouse TYK2 for PF-06673518 must be due to a difference in the amino acid sequence in the ATP binding site between human and mouse, and also other preclincal species. Examination of the amino acid sequence of the ATP binding sites of TYK2 in mouse, rat, dog, rhesus monkey, chimpanzee and human revealed complete homology between species with the exception of one residue (Fig. 2)^16^. This single residue is an isoleucine at position 960 (I960) in human TYK2, however the corresponding residue is a valine in mouse (V980), rat, dog, and cynomolgus monkey. Only the chimpanzee shares an isoleucine in common with humans (I960), calling into question the translational value of data generated in commonly used preclinical species.

**Figure 3:**
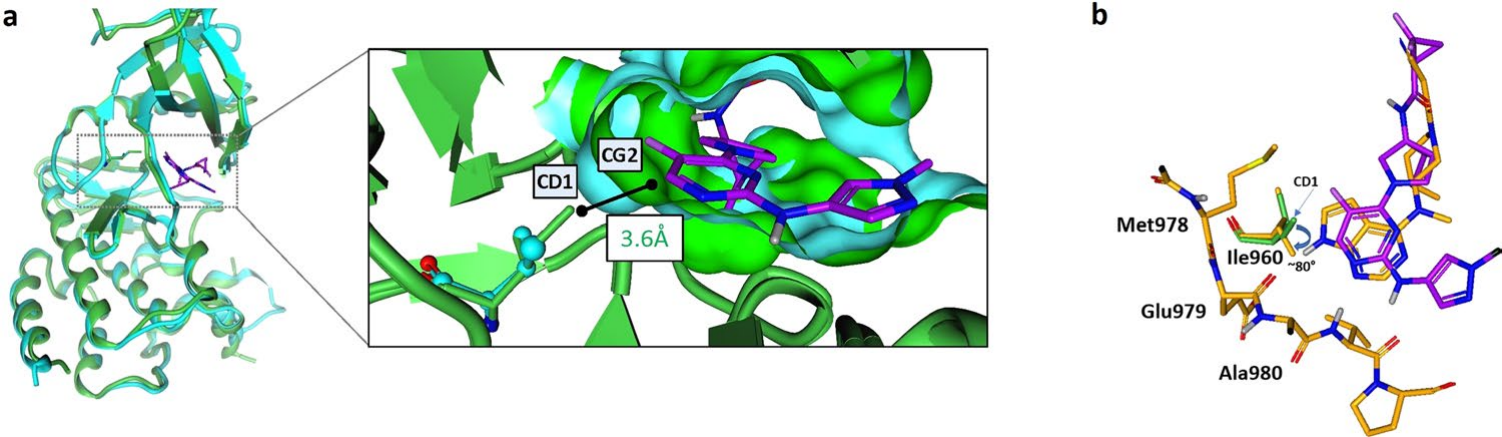
(**a**) Human TYK2 (Green) with PF-06673518 (Purple) from PDB code: 6VNX with isoleucine-960 overlaid with Mouse TYK2 protein structure (PDB code: 4E20) (Blue) with valine-980. Van der Walls surface of the two binding sites shown as solid surface; (**b**) Human TYK2 with Tofacitinib (orange) from PDB code: 3LXP verse PF-06673518 (purple) (PDB code: 6VNX) binding mode to hinge amino acids and Ile-960 in Human TYK2.

The protein X-ray structures for the human and mouse TYK2 JH1 domains were compared to assess the potential impact of this single amino acid variation^17^. The co-crystal structure of PF-06673518 with human TYK2 was obtained (PDB code 6VNX)^12^ and reveals a close interaction of the inhibitor with the CD1 methyl of isoleucine-960 (Fig. 3a). The distance between I960 CD1 and the C-6 carbon atom of the pyrimidine core is 3.6 Å, suggesting an energetically favorable hydrophobic protein-ligand interaction, within van der Waal radii^18^. Overlaying this structure with the published X-ray structure of mouse TYK2 kinase (4E20) using only the active site residues shows a highly aligned protein structure overall, yet illustrates a significantly larger distance of 4.9 Å (outside of sum of Van der Waal radii) between the mouse TYK2 valine-980 side-chain and CG2 carbon of the pyrimidine core of the inhibitor (see Supporting Information on Overlay technique). The loss of this lipophilic interaction due to the smaller valine side chain of mouse TYK2 is proposed to be the cause of the observed loss in potency between human and mouse. Previously published work by Pfizer on the characterization of tofacitinib in complex with the TYK2 kinase domain can rationalize why there is no loss of TYK2 potency between species^19^. In the structure of tofacitinib bound to TYK2 the pyrrolopyrimidine hinge binding core binds deeper into the ATP pocket than the aminopyrimidine series (Fig. 3b). This deeper binding pose forces the sidechain of I960 to fold down and away from tofacitinib. The I960 D1 carbon atom does not make significant contributions to the binding mode, which can in part explain why there is consistent potency of tofacitinib against TYK2 across species. From the structural analysis of TYK2 and the protein structure with PF-06673518, V980 in mouse TYK2 was hypothesized to be the key contributor to the loss in potency.

### Engineered I960V human protein and biochemical differences

We prepared a construct of the human TYK2 catalytic domain with a single amino acid change from wild-type (WT) isoleucine (I960) to valine (I960V) at position human 960. Inhibition of biochemical kinase activity was determined using the PerkinElmer LabChip EZ Reader (Caliper) assay platform with kinase constructs truncated to the catalytic domains^20^. The results indicated a good correlation between enzymatic kinase activity and cellular potency when the enzymatic functional assays were performed with ATP at a physiologically relevant concentration of 1 mM^21^. With the human TYK2 I960V mutant construct we can directly understand the impact of this single amino acid change. In accordance with our hypothesis, PF-06673518 showed a loss of potency (IC_50_ = 846 nM) in the human I960V mutant similar to what was observed in WT mouse TYK2 (IC_50_ = 1,407 nM). (Table 3) Tofacitinib in contrast demonstrated similar inhibition of TYK2, within 2-fold, across all three kinase constructs including the I960V mutant. This data supports the role of the valine residue as being primarily responsible for the loss in potency for PF-06673518 between mouse and human.

**Table 3:** Biochemical potency of tofacitinib^15^ and PF-06673518 TYK2 wild type human TYK2 kinase, mouse wild type TYK2, and human (I960V).

Compound	Human WT TYK2 IC_50_ (nM)	Mouse WT TYK2 IC_50_ (nM)	Human I960V TYK2 IC_50_ (nM)
Tofacitinib	489	966 ± 157	487 ± 89
PF-06673518	29 ± 11	1,407 ± 472	846 ± 284

Compounds were assayed at least three times, and the IC_50_ reported as the geometric mean ± standard deviation. ATP concentration = 1 mM.

**Figure 4:**
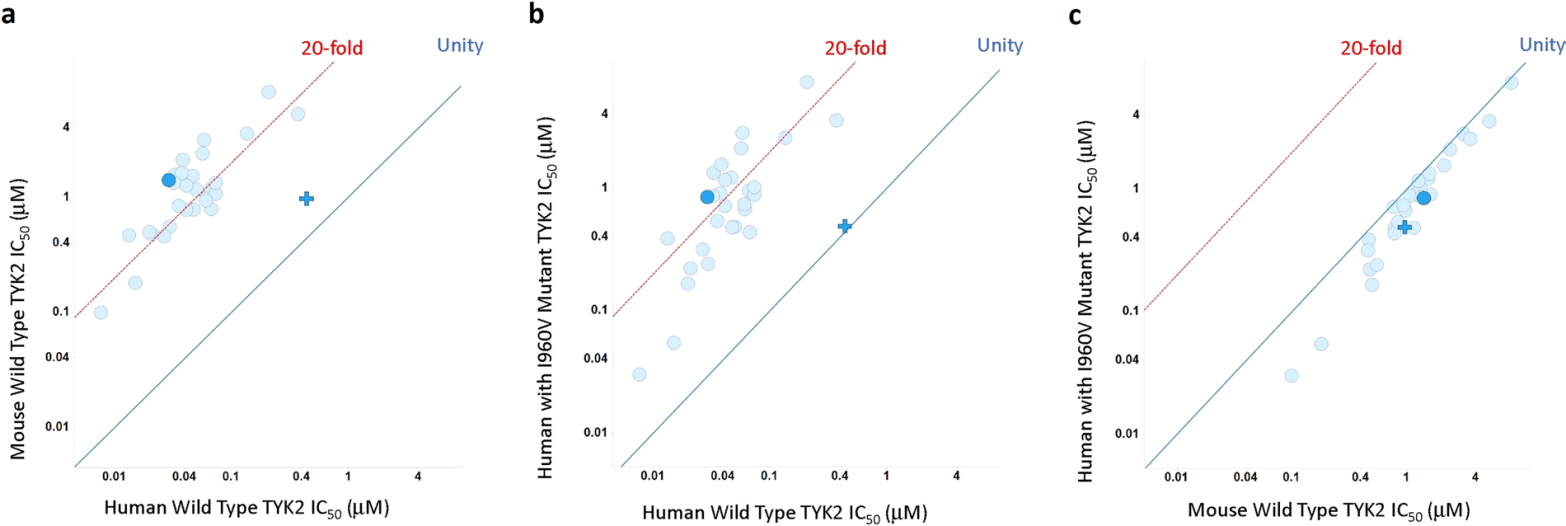
C orrelations for a group of 30 aminopyrimidines and tofacitinib: (**a**) scatter plot of human wild type TYK2 IC_50_ versus mouse wild type TYK2 IC_50_; (**b**) scatter plot of human wild type TYK2 IC_50_ versus human I960V mutant TYK2 IC_50_; (**c**) scatter plot of WT mouse TYK2 IC_50_ versus human I960V (mouse knock-in) TYK2 IC_50_. Tofacitinib dark blue (**+**); PF-06673518 dark blue (•). Compounds were assayed at least twice, and the IC_50_ reported as the geometric mean. ATP concentration = 1 mM.

To understand if this was a consistent issue across the aminopyrimidine series, a group of aminopyrimidine analogs of PF-06673518 with a range of potencies (IC_50_ ≈ 10–400 nM) against human TYK2 were selected for screening (See Supporting Information Table 1). The series of 30 analogs were uniformly 15- to 30-fold less potent in the mouse WT TYK2 assay than in the human WT TYK2 assay (Fig. 4a). The same trend was also apparent when comparing human I960V TYK2 to WT human TYK2 (Fig. 4b). By contrast, the biochemical potencies in WT mouse TYK2 and I960V human TYK2 were well-aligned near unity (Fig. 4c). For the aminopyrimidine series, we can therefore attribute the consistent loss of biochemical potency in WT mouse TKY2 to this single amino-acid difference, which could increase the challenge of interpreting results from *in vivo* experiments.

### Mutant mouse (V980I) cellular and *in vivo* data

We next developed a preclinical *in vivo* model that may more closely represented the expected human target^22^. In the mouse TYK2 protein, the homologous position to human isoleucine-960 (ref NP_003322.3) is valine-980 (ref NP_001192241 isoform 1). We therefore prepared a knock in mouse strain carrying this TYK2 V980I mutation. Before initiating the *in-vivo* study, lymphocytes from the V980I mice were isolated to confirm the effect of the knock-in mutation. In a similar manor as previously discussed, the TYK2 dependent IL-12 induced pSTAT4 inhibition was determined for tofacitinib and PF-06673518. Based on the previous results we expected no change in IC_50_ for tofacitinib, in contrast PF-06673518 was expected to be more potent on the V980I mutant compared to the WT mouse (Table 4). Tofacitinib showed inhibition of mouse V980I of 325 nM which was in line with human (IC_50_ = 145 nM) and in line with WT mouse (IC_50_ = 257 nM). In the V980I mouse IL-12 assay PF-06673518 had an IC_50_ = 128 nM, within 2-fold of the human WT and more potent than the mouse WT results (518 nM). With this data in hand, the TYK2 V980I mutant mouse appeared to be a more promising *in-vivo* model for testing this series of TYK2 inhibitors.

**Table 4:** Phospho-STAT inhibition in lymphocyte assays with mouse V980I Knock-in.

Compound	IL-12 (TYK2/JAK2) induced pSTAT4 IC_50_ (nM)		
	Human WT	Mouse WT	Mouse V980I KI^1^
Tofacitinib	145 ± 34	257 ± 34	325 ± 15
PF-06673518	64.3 ± 10.0	518 ± 84	128 ± 11

^1^Values represent mean IC_50_ (nM) ± standard deviation from two experiments.

**Figure 5:**
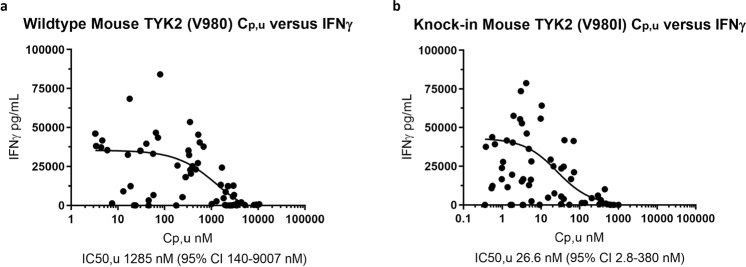
Effect of PF-06673518 to inhibit IL-12/IL-18 induced IFNγ production in (**a**) C57BL/6 wild type mice and (**b**) C57BL/6 TYK2 humanized V980I mice.

In the V980I knock-in mouse, we tested the ability of the TYK2/JAK1 inhibitor PF-06673518 to inhibit IFNγ production as a consequence of dual stimulation by both IL-12 and IL-18^23–25^. Wild type C57BL/6 and C57BL/6 TYK2 V980I mutant mice were administered PF-06673518 orally by gavage, followed 30 minutes later by a dual challenge with IL-12 and IL-18 cytokines. After 4 hours, the concentrations of IFNγ in the serum and of PF-06673518 in the plasma were measured. As shown in Fig. 5 the ability of PF-06673518 to inhibit IL-12/IL-18-induced IFNγ production was greater in C57BL/6 TYK2 V980I mice (IC_50_ = 26.6 nM) when compared to humanized C57BL/6 wild type mice (IC_50_ = 1,285 nM). These results indicate that PF-06673518 inhibits TYK2 at a lower concentration in the C57BL/6 V980I strain compared to the C57BL/6 wild type mouse. This outcome aligned with our results from biochemical and cellular assays, together showing that the potency of this series of TYK2 inhibitors is indeed affected by the single amino-acid difference between mouse and other preclinical species and human.

## Conclusion

During our TYK2 development program, we discovered that our lead series of aminopyrimidine TYK2 inhibitors was sensitive to a single amino-acid residue, isoleucine-960 in human TYK2, which makes a productive hydrophobic contact with the molecule. In standard preclinical species, bearing a valine at the analogous position in TYK2, this hydrophobic contact is missing, and as a consequence the compounds lost roughly 10 to 30-fold in potency. We prepared a humanized V980I TYK2 knock in mouse where we saw restoration of potency in primary cells and *in vivo*, relative to the wild type mouse. The cross-species potency shift illustrates the importance of understanding the translational pharmacology of the target from human to preclinical species early in the discovery process. Without this understanding of the molecular properties leading to species potency shifts between human and mouse TYK2, it could rationally have been concluded that the lead series of compounds had insufficient potency to achieve the required level of target modulation. In addition, a productive series of TYK2 inhibitors would have been erroneously abandoned without understanding cross-species translation. In contrast, toxicology studies with this chemical series would effectively characterize any TYK2-mediated liabilities for human risk assessment, since doses and exposures in animals typically will be high enough to supersede species differences in biochemical and cellular potency. This work supports the use of this humanized murine knock-in TYK2 strain for pharmacological studies for any series of TYK2 inhibitors where a close interaction with I960 is important to the binding mode and potency of the molecule.

## Methods

All activities involving laboratory animals were carried out in accordance with federal, state, local, and institutional guidelines governing the use of laboratory animals in research and were reviewed and approved by Pfizer (or other) Institutional Animal Care and Use Committee. Pfizer animal care facilities that supported this work are fully accredited by AAALAC International. All individuals blood donors gave their informed consent for the study (Pfizer Inc) and all methods were performed in accordance with the relevant guidelines and regulations. All studies was carried out according to the principles of the Declaration of Helsinki revised in 2013 for investigation with humans^26^.

### JAK inhibitors

Tofacitinib is commercially available from Sigma-Aldrich (CAS: 540737-29-9) and PF-06673518 along with other analogs were synthesized by Pfizer, Inc. Compounds used in biological studies had purities that were >95%, determined by HPLC, UPLC, or LC/MS based on ultraviolet detection at 220 or 254 nm (see Supporting Information). For *in vitro* assays, the compounds were prepared as 30 mM stocks in 100% dimethyl sulfoxide (DMSO). An 11-point dilution series was created in DMSO with a maximum concentration of 10 mM.

### JAK enzymes

GST-tagged recombinant human kinase domains of JAK1, JAK2, and JAK3 were purchased from Invitrogen. His-tagged recombinant human TYK2, mouse TYK2, and human TYK2 I960V mutant kinase domains were expressed in insect cell/baculovirus systems and purified using a two-step affinity and size exclusion purification method^13^.

### JAK caliper assay overview

Human Janus kinase (JAK) activity was determined using a microfluidic assay to monitor phosphorylation of a synthetic peptide by the recombinant human kinase domain of each of the four members of the JAK family, JAK1, JAK2, JAK3, and TYK2. Reaction mixtures contained 1 μM of a fluorescently labeled synthetic peptide and 1 mM ATP. Each assay condition was optimized for enzyme concentration and room temperature incubation time to obtain a conversion rate of 20 − 30% phosphorylated peptide product. Reactions were terminated by the addition of stop buffer containing EDTA. Utilizing mobility shift technology (PerkinElmer LabChip EZ Reader), each assay reaction was sampled to determine the level of phosphorylation. This technology is separation-based, allowing direct detection of fluorescently labeled substrates and products with separations controlled by a combination of vacuum pressure and electric field strength optimized for the peptide substrate. See the Supporting Information for commercial and sequence information^13,21^.

### Caliper JAK enzyme end-point IC50 assays

Test compounds were solubilized in dimethyl sulfoxide (DMSO) to a stock concentration of 30 mM. Compounds were diluted in DMSO to create an 11-point half-log dilution series with a top concentration of 600 μM. The test compound plate also contained positive control wells with a known inhibitor to define 100% inhibition and negative control wells with DMSO to define no inhibition. The compound plates were diluted 1 to 60 in the assay, resulting in a final assay compound concentration range of 10 μM to 100 pM and a final assay concentration of 1.7% DMSO. Two hundred fifty nanoliters of test compounds and controls solubilized in 100% DMSO were added to a 384-well polypropylene plate using a non-contact acoustic dispenser. Kinase assays were carried out at room temperature in a 15 μL reaction buffer containing 20 mM HEPES, pH 7.4, 10 mM magnesium chloride, 0.01% bovine serum albumin (BSA), 0.0005% Tween20, 1 mM DTT and 1 mM ATP. Reaction mixtures contained 1 μM of a fluorescently labeled synthetic peptide, a concentration lower than the apparent Km (5FAM-KKSRGDYMTMQID for JAK1 and TYK2 and FITC-KGGEEEEYFELVKK for JAK2 and JAK3). The assays were stopped with 15 μL of a buffer containing 180 mM HEPES, pH 7.4, 20 mM EDTA, and 0.2% coating reagent, resulting in a final concentration of 10 mM EDTA, 0.1% coating reagent, and 100 mM HEPES, pH 7.4. Each assay reaction was then sampled to determine the level of phosphorylation. The data output used for calculations was percent product converted and was determined for each sample and control well based on peak height (percent product = product/ (product + substrate)). The percent effect at each concentration of test compound was calculated based on the positive and negative control well contained within each assay plate using the following formula: percent effect = 100((sample well − negative control)/(positive control − negative control)). The percent effect was plotted against the compound concentration compound. An unconstrained sigmoid curve was fitted using a four-parameter logistic model, and the concentration of test compound required for 50% inhibition (IC50) was determined for each test compound.

### Reagents

Cytokines human IFNα (Catalog No. 11200-2), monkey IFNα (Catalog No. 14110-1), mouse IFNα (Catalog No. 12100-1), humanIL-12 (Catalog No. 219-IL), monkey IL-12 (Catalog No. 3216-RL), dog IL-12 (Catalog No. 2118-CL), mouse IL-12 (Catalog No. 419-ML), human IL-15 (Catalog No. 247-IL) were obtained from R&D Systems (Minneapolis, MN, USA). Dog IFNα (Catalog No. AB1697202) was purchased from Abcam (Cambridge, UK). Antibodies specific to phosphorylated signal transducer and active of transcription proteins (pSTATs) were supplied by BD Biosciences (San Jose, CA, USA): Anti-pSTAT1-AF488 (Catalog No. 612596); Anti-pSTAT3-AF647 (Catalog No. 557815); Anti-pSTAT4-AF647 (Catalog No. 558137); Anti-pSTAT5-AF647 (Catalog No. 612599). Phosflow Lyse/Fix Buffer 5×(Catalog No. 558049) and BD Pharm Lyse lysing buffer (Catalog No. 555899) were purchased from BD Biosciences. Fetal bovine serum (Catalog No. A3160601) was purchased from Thermo Fisher Scientific (Waltham, MA, USA) and sodium azide (Catalog No. S8032) was obtained from Sigma Aldrich (St. Louis, MO, USA). D-PBS (Catalog No. 14190) and RPMI1640 medium (Catalog No. 11875-093) were obtained from Invitrogen (Grand Island, NY, USA).

### Inhibition of cytokine-induced phosphorylation of STATs by JAK inhibitors

Human blood samples were collected from healthy donors via vein puncture in accordance with Pfizer protocols (Protocol No. GOHW RDP-01) approved by the Shulman Institutional Review Board. Monkey (cynomolgus), dog (Beagle), and mouse (C57BL/6) blood samples were purchased from BioIVT (Westbury, NY, USA). C57BL/6 TYK2 V980I knock-in mice were generated for Pfizer by Taconic Biosciences (Hudson, NY, USA). TYK2 V980I knock-in mouse blood samples were prepared at the study site (Cambridge, MA, USA). Heparin was used as the anticoagulant for all blood samples. Blood was treated with BD Pharm Lyse lysing buffer to remove erythrocytes. The remaining leukocytes were washed once with D-PBS and suspended in RPMI1640 medium containing 10% fetal bovine serum. Cells were incubated at 37 °C for 2 hours. The resting leukocytes were resuspended in RPMI1640 medium without serum or other proteins. Leukocytes were aliquoted (90 μL/well) in 96-well, deep-well, V-bottom plates and treated with tofacitinib or PF-06673518 (5 μL/well) at various concentrations (0.3 nM to 20 µM) at 37 °C for 60 minutes. This was followed by a challenge with cytokine (5 μL/well; final, 5 ng/mL IL-12, or 30 ng/mL IL-15) for 15 minutes. Samples were treated with warm 1X Lyse/Fix buffer (700 μL/well) to terminate activation and further incubated at 37 °C for 20 minutes to fix cells. Plates were centrifuged at 300 x g for 5 minutes, supernatant was aspirated, and cells were washed with 600 μL per well of staining buffer (D-PBS containing 0.1% fetal bovine serum and 0.01% sodium azide). Washed cell pellets were suspended with 350 μL per well of pre-chilled 90% methanol and incubated on ice for 30 minutes. After the removal of 90% methanol, cells were washed once with staining buffer (600 μL/well). Cell pellets were suspended in staining buffer containing fluorophore conjugated anti-phospho-STAT antibodies (1 to 120 dilution, 120 μL/well), and incubated at 4 °C in the dark overnight. Anti-pSTAT4-AlexaFluor647 was used for IL-12-stimulated samples. Anti-pSTAT5-AlexaFluor647 was used for IL-15 stimulated samples^21,27^.

### Flow cytometry

Samples were transferred to 96-well U-bottom plates and flow cytometric analysis was performed on an LSRFortessa equipped with a HTS plate loader (BD Biosciences). The lymphocyte population was gated for histogram analysis of phospho-STAT staining. Background fluorescence was defined using unstimulated cells and a gate was placed at the foot of the peak to include ~0.5% gated population. The histogram statistical analysis was performed using FACSDiva version 8.0.1 (BD Biosciences) software. Relative fluorescence unit (RFU), which measures the level of phospho-STAT, was calculated by multiplying the percent positive population and its mean fluorescence. Data from 11 compound concentrations (singlicate at each concentration) was used to determine IC_50_ values using the Prism version 8 software (GraphPad, La Jolla, CA, USA)^21^.

### Generation of humanized TYK2 V980I Mouse (C57BL/6-Tyk2 < tm3672.1(V980I)Arte > )

The quality-tested ES cell line (ART B6 3.6, genetic background: C57BL6/N Tac) was grown on a mitotically inactivated feeder layer comprised of mouse embryonic fibroblasts in ES cell culture medium containing Leukemia inhibitory factor and Fetal Bovine Serum. The cells were electroporated with the linearized DNA targeting vector according to Taconic Standard Operation Procedures. The selection mechanisms used are Puromycin and Ganciclovir selection. Resistant ES cell clones with a distinct morphology were isolated and analyzed by PCR in a primary screen and validated by Southern Blotting and PCR analysis. Homologous recombinant ES cell clones were expanded and frozen in liquid nitrogen after extensive molecular validation.

### Detection of the inserted point mutation (V980I)

#### PCR Primers

10484_1_Tyk2_525: AACACTCAGGAGGCAGAGGCAAGTGC

10484_2_Puro_F2: GTGCCTGAACCGGTTCGAGATCCAG

Expected Fragments [bp]: 1476(targ)

Expected Control Band [bp]: 585(ctrl)

Probe information for Southern Blot Analysis: Primer sequences for the PCR amplification of the Tyk2 5ext2 probe: Sense: ctatccagtgccacccacactg, Antisense: gactggggtcgagaactttgag

Primer sequences for the PCR amplification of the Tyk2 3ext1 probe: Sense: gggtgactttctaatgctgatg, Antisense: taaacaagtgatgagcttcgtg

After administration of hormones, super ovulated BALB/c females were mated with BALB/c males. Blastocysts were isolated from the uterus at dpc 3.5. For microinjection, blastocysts were placed in a drop of DMEM with 15% FCS under mineral oil. A flat tip, piezo actuated microinjection-pipette with an internal diameter of 12–15 micrometer was used to inject 10-15 targeted C57BL/6NTac ES cells into each blastocyst. After recovery, 8 injected blastocysts were transferred to each uterine horn of 2.5 days post coitum, pseudopregnant NMRI females. Chimerism was measured in chimeras (G0) by coat color contribution of ES cells to the BALB/c host (black/white). Highly chimeric mice were bred to strain C57BL/6 females. To generate humanized C57BL/6, mice were mated with B6 Flp-Deleter TG mice for *in-vivo* selection marker deletion and to generate mice heterozygous for the humanized knock-in allele. Germline transmission was identified by the presence of black, strain C57BL/6, offspring (G1).

#### Genotyping analysis

Genomic DNA was extracted from tail biopsies and analyzed by PCR. The following templates were used as controls: H_2_O (ctrl1), wildtype genomic DNA (ctrl2), positive DNA sample (ctrl3). The amplification of the internal control fragment - 585 bp (ctrl) - with oligos 1260_1 and 1260_2 confirms the presence of DNA in the PCR reactions (amplification of the CD79b wildtype allele, nt 17714036-17714620 on Chromosome 11). The PCR amplicons were analyzed by using a Caliper LabChip GX device. The transgenic strain of mice, C57BL/6-Tyk2 < tm3672.1(V980I)Arte> with the humanized TYK2, is referred to as TYK2 V980I.

#### Animals

Male TYK2 V980I as well as wild-type C57BL/6 mice were generated by and obtained from Taconic Farms Germantown, NY. All mice were housed at least 3 days prior to the study in a specific pathogen-free environment at a Pfizer animal facility with ad libitum access to food and purified water. Animals were used between 8 and 16 weeks of age. All studies were performed in accordance with American Association for the Accreditation of Lab Animal Care animal welfare standards and under Internal Animal Care and Use Committee approval.

#### PK/PD IL-12/18 induced interferonγ production model

C57BL/6 TYK2 V980I transgenic mice and wild type C57BL/6 mice were dosed orally with either vehicle or PF-06673518 at doses from 3 to 100 mg/kg. The vehicle used in all cases was 0.5% methylcellulose, 2% Tween-80 in water. 30 minutes later mice were injected intraperitoneally with 200 µL of a cocktail of 0.02 µg IL-12 (R&D systems) and 2 µg IL-18 (MBL International). Four hours later mice were euthanized and bled by cardiac puncture. Plasma was collected to measure exposures using standard LC-MS/MS methods and serum was collected to quantify IFNγ via ELISA (R&D Systems). The resultant values of IFNγ and exposure were graphed in GraphPad Prism version 8.0, and IC_50_ values were calculated by using a three-parameter nonlinear fit.

## Supplementary information

Supplementary Information.
