# Supplementary material for: Demonstration of In Vitro to In Vivo Translation of a TYK2 Inhibitor That Shows Cross Species Potency Differences
Source: Sci Rep. 2020 Jun 2;10:8974. doi: 10.1038/s41598-020-65762-y (PMC7265552; doi:10.1038/s41598-020-65762-y)
Supplement: Supplementary file 1 — Supplementary Information. [file 41598_2020_65762_MOESM1_ESM.docx]

**Demonstration of In Vitro to In Vivo Translation of a TYK2 Inhibitor That Shows Cross Species Potency Differences**

# Brian S. Gerstenberger^1,*^ Mary Ellen Banker^2^, James D. Clark^3^, Martin E. Dowty^1^, Andrew Fensome^1^, Roger Gifford^3^, Matthew C. Griffor^2^, Martin Hegen^3^, Brett D. Hollingshead^4^, John D. Knafels^2^, Tsung H. Lin^3^, James F Smith^2^, and Felix F. Vajdos^2^

^1^Medicine Design, Pfizer Inc, 1 Portland Street, Cambridge, Massachusetts 02139, United States

^2^Medicine Design, Pfizer Inc, Eastern Point Road, Groton, Connecticut 06340, United States

^3^Inflammation and Immunology, Pfizer Inc, 1 Portland Street, Cambridge, Massachusetts 02139, United States

^4^Drug Safety Research and Development, Pfizer Inc., 1 Portland Street, Cambridge, Massachusetts, 02139, United States

^*^brian.gerstenberger@pfizer.com

**Structural Alignment:** The superposition is a least-squares fit between the c-alpha atoms of 9 active site residues in each kinase (hTyk2 residues I960, K961, M978, E979, Y980, P982, G984, S985, N1028; mTyk2 residues V956, K957, M974, E975, Y976, P978, G980, S981, N1024).

**SI Table 1:** TYK2 Data and Structures of Compounds in Figure 3 IC_50_ reported as the geometric mean ± standard deviation.

| Compound  Number | Structure | Human TYK2 I960V IC_50_ (uM) | Mouse WT TYK2 IC_50_ (uM) | Human WT TYK2 IC50 IC_50_ (uM) |
| --- | --- | --- | --- | --- |
| 1 |  | 0.316 ± 0.149 | 0.459 ± 0.139 | 0.027 ± 0.007 |
| 2 |  | 0.166 ± 0.035 | 0.495 ± 0.165 | 0.020 ± 0.007 |
| 3 |  | 0.882 ± 0.159 | 1.074 ± 0.128 | 0.073 ± 0.017 |
| 4 |  | 0.738 ± 0.021 | 0.935 ± 0.060 | 0.06 ± 0.018 |
| 5 |  | 0.716 ± 0.250 | 0.77 ± 0.219 | 0.041 ± 0.013 |
| 6 |  | 0.48 ± 0.182 | 0.781 ± 0.313 | 0.047 ± 0.021 |
| 7 |  | 0.673 ± 0.071 | 0.972 ± 0.120 | 0.061 ± 0.023 |
| 8 |  | 0.846 ± 0.284 | 1.407 ± 0.472 | 0.029 ± 0.011 |
| 9 |  | 0.858 ± 0.122 | 1.335 ± 0.156 | 0.032 ± 0.014 |
| 10 |  | 0.221 ± 0.012 | 0.474 ± 0.039 | 0.021 ± 0.001 |
| 11 |  | 0.951 ± 0.379 | 1.161 ± 0.304 | 0.066 ± 0.028 |
| 12 |  | 2.1 21 ± 0.145 | 2.387 ± 0.094 | 0.057 ± 0.018 |
| 13 |  | 0.241 ± 0.067 | 0.551 ± 0.116 | 0.029 ± 0.009 |
| 14 |  | 0.903 ± 0.033 | 1.62 ± 0.257 | 0.037 ± 0.027 |
| 15 |  | 3.591 ± 0.257 | 5.244 ± 0.605 | 0.371 ± 0.037 |
| 16 |  | 2.572 ± 1.559 | 3.577 ± 1.983 | 0.135 ± 0.089 |
| 17 |  | 1.225 ± 0.220 | 1.524 ± 0.217 | 0.046 ± 0.011 |
| 18 |  | 0.538 ± 0.622 | 0.841 ± 0.989 | 0.035 ± 0.034 |
| 19 |  | 7.389 ± 1.953 | 8.163 ± 2.027 | 0.208 ± 0.051 |
| 20 |  | 0.435 ± 0.174 | 0.784 ± 0.207 | 0.067 ± 0.021 |
| 21 |  | 0.484 ± 0.369 | 1.158 ± 1.037 | 0.05 ± 0.046 |
| 22 |  | 1.017 ± 0.263 | 1.327 ± 0.396 | 0.073 ± 0.018 |
| 23 |  | 0.388 ± 0.050 | 0.466 ± 0.078 | 0.013 ± 0.004 |
| 24 |  | 1.335 ± 0.386 | 1.569 ± 0.506 | 0.033 ± 0.011 |
| 25 |  | 2.824 ± 0.631 | 3.13 ± 0.832 | 0.058 ± 0.006 |
| 26 |  | 1.564 ± 0.257 | 2.117 ± 0.465 | 0.038 ± 0.008 |
| 27 |  | 1.173 ± 0.082 | 1.259 ± 0.113 | 0.041 ± 0.006 |
| 28 |  | 0.055 ± 0.025 | 0.180 ± 0.089 | 0.015 ± 0.006 |
| 29 |  | 0.03 ± 0.001 | 0.100 ± 0.017 | 0.008 ± 0.004 |

**Synthesis and Characterization of Compounds in SI Table 1.**

Synthesis and characterization of compound 1-11 was previously described in WO16027195. Synthesis and characterization of compounds 12 and 13 was previously described in Journal of Medicinal Chemistry 61, 8597-8612, <https://doi.org/10.1021/acs.jmedchem.8b00917>. Synthesis and characterization of compound 14 was previously described in A. Fensome, et al. “Design and Optimization of a Series of 4-(3-azabicyclo[3.1.0]hexan-3-yl)pyrimidin-2-amines: Dual Inhibitors of TYK2 and JAK1” Bioorganic & Medicinal Chemistry , 2020, <https://doi.org/10.1016/j.bmc.2020.115481>.

**N-ethyl-4-((5-methyl-4-((1R,4R)-5-(methylsulfonyl)-2,5-diazabicyclo[2.2.1]heptan-2-yl)pyrimidin-2-yl)amino)benzamide (Compound 15):** Synthesized in a similar manner to compound 11 in JMC 2018, 11, 8597-8612 to provide 103 mg. MS m/z = 430.9 [M+H]^+^; HPLC RT = 2.91 min, purity 95.86% [Waters Symmetry 2.1x50 mm, 0% MeCN in water(0.1% TFA) to 60% over 8 mins]; ^1^H NMR (400 MHZ, DMSO-d_6_) δ: 1.12 (m, 3H), 1.99 (m, 2H), 2.17 (s, 3H), 3.01 (s, 3H), 3.27 (m, 2H), 3.55 (m, 2H), 3.84 (dd, J=2Hz, 1H), 3.87 (dd, J=2Hz, 1H), 4.47 (s, 2H), 4.98 (s, 1H), 7.78 (m, 4H), 7.84 (s, 1H), 8.23 (m, 1H), 9.30 (s, 1H).

**N-ethyl-4-((4-((1R,5S)-6-(2,2,2-trifluoroacetyl)-3,6-diazabicyclo[3.1.1]heptan-3-yl)pyrimidin-2-yl)amino)benzamide (Compound 16):** Synthesized in a similar manner to compound 114 in WO16027195 to provide 12 mg. MS m/z = 435.2 [M+H]^+^; HPLC RT = 1.56 min, purity = 94% [Waters Acquity HSS T3, 2.1mmx50mm, C18, 1.7µm; 0.1% formic acid in water (v/v); Mobile phase B: 0.1% formic acid in acetonitrile (v/v).Initial conditions: A-95%:B-5%; hold at initial from 0.0-0.1min; Linear Ramp to A-5%:B-95% over 0.1-1.0min; hold at A-5%:B-95% from 1.0-1.1min]; ^1^H NMR (600 MHZ, DMSO-d_6_) δ: 1.16 (t, J=7.0 Hz, 3 H) 1.85 (d, J=9.2 Hz, 1 H) 2.93 - 3.01 (m, 1 H) 3.26 - 3.36 (m, 2 H) 3.87 - 4.19 (m, 4 H) 4.80 (bs, 1 H) 5.04 (bs, 1 H) 6.50 (bs, 1 H) 7.78 (bs, 2 H) 7.91 (d, J=7.0 Hz, 2 H) 8.15 (d, J=6.2 Hz, 1 H) 8.41 (bs, 1 H) 10.50 (bs, 1 H).

**5-((4-((1R,5S)-8-(2-cyanoethyl)-3,8-diazabicyclo[3.2.1]octan-3-yl)-5-fluoropyrimidin-2-yl)amino)-N-ethyl-3-methylpicolinamide (Compound 17):** Synthesized in a similar manner to compound 80 in WO16027195 to provide 47 mg. MS m/z = 439.2 [M+H]^+^; HPLC RT = 3.32 min, purity = 99.35% [Ultimate XB-C18 3 pm x 350 mm; 0% MeCN in water (0.1% TFA) to 60% over 6 mins]; ^1^H NMR (400MHz, MeOD-d_4_) δ: 1.2 (t, J=7.6 Hz, 3H), 1.71-1.79 (m, 2H), 1.97-1.99 (m, 2H), 2.58-2.74 (m, 4H), 3.20-3.26 (m, 2H), 3.29-3.47 (m, 4H), 4.19 (d, J=10.8 Hz, 1H), 7.88 (d, J=7.2 Hz, 1H), 7.99 (d, J=2 Hz, 1H), 8.67 (d, J=2 Hz, 1H).

**5-((5-chloro-4-((1S,5R)-1-(cyclopropanecarboxamido)-3-azabicyclo[3.1.0]hexan-3-yl)pyrimidin-2-yl)amino)-N-ethyl-3-methylpicolinamide (Compound 18):** Synthesized in a similar manner to compound 100 in WO16027195 to provide 23 mg. MS m/z = 456 [M+H]^+^; HPLC RT = 2.67 min, purity = 96.34% [Ultimate XB-C18 3 pm x 350 mm; 0% MeCN in water (0.1% TFA) to 60% over 6 mins]; ^1^H NMR (400 MHZ, DMSO-d6) δ:0.72-0.75 (m, 5H), 1.03 (m, 1H), 1.10 (t, J=7.2Hz, 3H), 1.52 (m, 1H), 1.75-1.77 (m, 1H), 2.53 (s, 3H), 3.29 (m, 2H), 3.70-3.73 (m, 1H), 3.86-3.90 (m, 1H), 4.07-4.10 (m, 1H), 4.32-4.35 (m, 1H), 8.04 (s, 1H), 8.09 (d, J=2 Hz, 1H), 8.46 (t, J=5.6 Hz, 1H), 8.66 (d, J=2Hz, 1H), 8.72 (s, 1H), 9.70 (s, 1H)

**5-((4-((1S,5R)-1-(cyclopropanecarboxamido)-3-azabicyclo[3.1.0]hexan-3-yl)pyrimidin-2-yl)amino)-N,3-dimethylpicolinamide (Compound 19):** Synthesized in a similar manner to compound 100 in WO16027195 to provide 20 mg. MS m/z = 408 [M+H]^+^; HPLC RT = 2.52 min, purity = 95.03% [Symmety C18 5 pm 2.1*50 mm, 0% MeCN in water (0.1% TFA) to 60% over 6 mins)]; ^1^H NMR (400 MHZ, DMSO-d_6_) δ: 0.66-0.71 (m, 5H), 1.05-1.07 (m, 1H), 1.49-1.52 (m, 1 H), 1.76 (m, 1H), 2.54 (s, 3H), 2.75 (d, J=4.8 Hz, 3H), 3.50-4.06 (m, 4H), 6.02 (d, J=6.0 Hz, 1H), 7.97 (d, J=6.0 Hz, 1H), 8.13 (m, 1H), 8.42 (d, J=4.4 Hz, 1H), 8.73 (s, 1H), 8.80 (s, 1H), 9.48 (s, 1H).

**N-((1S)-3-(2-((1-(2-hydroxyethyl)-1H-pyrazol-4-yl)amino)-5-methylpyrimidin-4-yl)-3-azabicyclo[3.1.0]hexan-1-yl)cyclopropanecarboxamide (Compound 20):** Synthesized in a similar manner to compound 107 in WO16027195 to provide 44 mg. MS m/z = 384.1 [M+H]^+^; HPLC RT = 3.42 min, purity = 97.36% [Ultimate XB-C18 3 pm x 350 mm; 0% MeCN in water (0.1% TFA) to 60% over 6 mins]; ^1^H NMR (400MHz, MeOD-d_4_) δ: 0.78-0.81 (m, 3H), 0.85-0.88 (m, 2H), 1.16-1.19 (m, 1H), 1.55-1.56 (m, 1H), 1.84-1.86 (m, 1H), 2.31 (s, 3H), 3.89-3.92 (m, 3H), 4.15 (m, 2H), 4.22-4.25 (m, 2H), 4.41-4.44 (m, 1H), 7.44 (s, 1H), 7.57 (s, 1H), 7.88 (s, 1H).

**N-((1S,5R,6R)-6-methyl-3-(5-methyl-2-((1-methyl-1H-pyrazol-4-yl)amino)pyrimidin-4-yl)-3-azabicyclo[3.1.0]hexan-1-yl)cyclopropanecarboxamide (Compound 21):** Synthesized in a similar manner to compound 93 in WO16027195 to provide 49 mg. MS m/z = 368.1 [M+H]^+^; HPLC RT = 2.44 min, purity = 97.22% [C18 3 um 2.1*30 mm; 0% MeCN in water (0.1% TFA) to 60% 6 min]; ^1^H NMR (400MHz, MeOD-d_4_) δ: 0.75-0.78 (m, 2H), 0.85 (m, 2H), 1.04 (d, J=6.8 Hz, 3H), 1.29-1.31 (m, 1H), 1.53 (s, 1H), 1.77-1.81 (m, 1H), 2.27 (s, 3H), 3.82-3.87 (m, 5H), 4.07-4.10 (m, 1H), 4.17-4.19 (m, 1H), 7.52 (s, 1H), 7.56 (s, 1H), 7.75 (s, 1H)

**3-((1R,5S)-3-(2-((1-methyl-1H-pyrazol-4-yl)amino)pyrimidin-4-yl)-3,8-diazabicyclo[3.2.1]octan-8-yl)propanenitrile (Compound 22):** Synthesized in a similar manner to compound 79 in WO16027195 to provide 24 mg. MS m/z = 339.2 [M+H]^+^; HPLC RT = 2.53 min, purity = 95.37% [Xbridge RP18 5 um 2.1*50mm, 10% MeCN in water (0.1% TFA) to 80% over 6 mins]; ^1^H NMR (400 MHZ, DMSO-d_6_) δ:1.24 (s, 1H), 1.50 (d, J=7.5 Hz, 2H), 1.85 (d, J=4.8 Hz, 2H), 2.57 - 2.63 (m, 2 H), 2.66 - 2.72 (m, 2H), 3.00 (d, J=11.5 Hz, 2H), 3.37 - 3.47 (m, 3H), 3.72 - 3.99 (m, 5H), 4.47 (s, 1H), 6.06 (d, J=6.3 Hz, 1H), 7.43 (s, 1H), 7.73 (bs, 1H), 7.88 (d, J=6.0 Hz, 1H), 8.83 (bs, 1H)

**(1R)-N-((1S,6R)-3-(2-((1H-pyrazol-4-yl)amino)-5-fluoropyrimidin-4-yl)-6-methyl-3-azabicyclo[3.1.0]hexan-1-yl)-2,2-difluorocyclopropane-1-carboxamide (Compound 23):** Synthesized in a similar manner to compound 94 in WO16027195 to provide 43 mg. MS m/z = 394.1 [M+H]^+^; HPLC RT = 0.72 min, purity = 99% [Waters Acquity HSS T3, 2.1mmx50mm, C18, 1.7µm; Column Temperature 60°C; 0.1% formic acid in water (v/v); Mobile phase B: 0.1% formic acid in acetonitrile (v/v).Initial conditions: A-95%:B-5%; hold at initial from 0.0-0.1min; Linear Ramp to A-5%:B-95% over 0.1-1.0min; hold at A-5%:B-95% from 1.0-1.1min]; ^1^H NMR (400MHz, MeOD-d_4_) δ: 0.93 (d, J=6.2 Hz, 3H), 1.00-1.16 (m, 3H), 1.22-1.29 (m, 1H), 1.62-1.69 (bs, 1H), 1.71-1.81 (m, , 1H), 1.83-2.08 (m, 1H), 2.28-2.45

**(1S)-N-((1S,6R)-3-(2-((1H-pyrazol-4-yl)amino)-5-fluoropyrimidin-4-yl)-6-methyl-3-azabicyclo[3.1.0]hexan-1-yl)-2,2-difluorocyclopropane-1-carboxamide (Compound 24):** Synthesized in a similar manner to compound 95 in WO16027195 to provide 53 mg. MS m/z = 394.2 [M+H]^+^; HPLC RT = 0.73 min, purity = 99% [Waters Acquity HSS T3, 2.1mmx50mm, C18, 1.7µm; Column Temperature 60°C; 0.1% formic acid in water (v/v); Mobile phase B: 0.1% formic acid in acetonitrile (v/v).Initial conditions: A-95%:B-5%; hold at initial from 0.0-0.1min; Linear Ramp to A-5%:B-95% over 0.1-1.0min; hold at A-5%:B-95% from 1.0-1.1min]; ^1^H NMR (400MHz, MeOD-d_4_) δ: 0.93 (d, J=6.63 Hz, 3H), 1.00 - 1.18 (m, 3H), 1.22-1.46 (m, 1H), 1.66 (br d, J=11.3 Hz, 1H), 1.75 (br dd, J=9.0, 5.5 Hz, 1 H), 1.84-1.98 (m, 2H), 2.36 (br dd, J=10.5, 2.7 Hz, 1H), 3.23-3.42 (m, 2 H), 3.68 (br dd, J=11.71, 2.73 Hz, H), 3.88-4.08 (m, 2H), 7.64-7.66 (m, 3H)

**(1R,2R)-2-cyano-N-((1S,5R)-3-(5-fluoro-2-((1-methyl-1H-pyrazol-4-yl)amino)pyrimidin-4-yl)-3-azabicyclo[3.1.0]hexan-1-yl)cyclopropane-1-carboxamide (Compound 25):** Synthesized in a similar manner to compound 95 in WO16027195 to provide 23 mg. MS m/z = 393 [M+H]^+^; HPLC RT = 2.34 min, purity = 98.5% [Ultimate XB-C18 3 pm 3x50 mm, 0% MeCN in water (0.1% TFA) to 60% MeCN over 6 min]; ^1^H NMR (400 MHZ, DMSO-d_6_) δ: 0.78 (s, 1H), 1.04-1.11 (m, 1H), 1.17 (s, 1H), 1.25-1.32 (m, 1 H), 1.46 (d, J=4.3 Hz, 1 H), 1.80 (bs, 1H), 1.99 (s, 1H), 2.17-2.28 (m, 1 H), 3.57-3.63 (m, 1H), 3.75-3.84 (m, 5H), 4.06-4.13 (m, 1H), 7.41 (s, 1H), 7.72 (s, 1H), 7.89 (d, J=6.3 Hz, 1H), 8.91-9.06 (m, 1H), 9.10 (s, 1H)

**(S)-2,2-difluoro-N-((1S,5R)-3-(5-fluoro-2-((1-methyl-1H-pyrazol-4-yl)amino)pyrimidin-4-yl)-3-azabicyclo[3.1.0]hexan-1-yl)cyclopropane-1-carboxamide (Compound 26):** Synthesized in a similar manner to compound 93 in WO16027195 to provide 36 mg. MS m/z = 394.1 [M+H]^+^; HPLC RT = 2.39 min, purity = 99.01% [Ultimate XB-C18 3 pm 3x50 mm, 0% MeCN in water (0.1% TFA) to 60% MeCN over 6 min]; ^1^H NMR (400 MHZ, DMSO-d_6_) δ: 0.76-0.81 (m, 1H), 0.99-1.08 (m, 1H), 1.76-1.95 (m, 3H), 2.52-2.60 (m, 1H), 3.54-3.61 (m, 1H); 3.76 (s, 5H), 4.04-4.12 (m, 1H), 7.40 (s, 1H), 7.71 (s, 1H), 7.88 (d, J=6.0 Hz, 1H), 8.88-8.96 (m, 1H), 8.99 (s, 1H)

**(1R,2R)-2-cyano-N-((1S,5R,6R)-3-(5-fluoro-2-((6-(2-hydroxyethyl)-5-methylpyridin-3-yl)amino)pyrimidin-4-yl)-6-methyl-3-azabicyclo[3.1.0]hexan-1-yl)cyclopropane-1-carboxamide (Compound 27):** Synthesized in a similar manner to compound 98 in WO16027195 to provide 31 mg. MS m/z = 452 [M+H]^+^; HPLC RT = 0.95 min, purity = >99% [Ultimate XB-C18 2.1*30 mm; 0% MeCN in water (0.1% TFA) to 60% over 2 mins)]; ^1^H NMR (400 MHZ, DMSO-d_6_) δ: 0.93 (d, J=6.5 Hz, 3H), 1.31 (d, J=9.0 Hz, 2H), 1.42-1.51 (m, 1H), 1.81-1.88 (m, 1H), 1.95-2.03 (m, 1H), 2.25 (s, 4H), 2.82 (s, 2H), 3.62-3.74 (m, 4H), 3.88-3.99 (m, 2H), 4.63 (s, 1H), 7.90 (bs, 1H), 7.96 (d, J=6.0 Hz, 1H), 8.58 (bs, 1 H), 9.13 (s, 1 H), 9.17 (s, 1 H)

**((S)-2,2-difluorocyclopropyl)((1R,5S)-3-(5-methyl-2-((1-methyl-1H-pyrazol-4-yl)amino)pyrimidin-4-yl)-3,8-diazabicyclo[3.2.1]octan-8-yl)methanone (Compound 28):** Synthesized in a similar manner to compound 7 in WO16027195 to provide 103 mg. MS m/z = 404.3 [M+H]^+^; HPLC RT = 0.57 min, purity = >99% [Waters Acquity HSS T3, 2.1mmx50mm, C18, 1.7µm; Column Temperature 60°C; 0.1% formic acid in water (v/v); Mobile phase B: 0.1% formic acid in acetonitrile (v/v).Initial conditions: A-95%:B-5%; hold at initial from 0.0-0.1min; Linear Ramp to A-5%:B-95% over 0.1-1.0min; hold at A-5%:B-95% from 1.0-1.1min]; ^1^H NMR (400MHz, MeOD-d_4_) δ: 1.83 (br dd, J=11.51, 6.83 Hz, 1H) 1.94 - 2.21 (m, 9 ) 2.99-3.27 (m, 3H) 3.86-4.08 (m, 6H) 4.57-4.67 (m, 1H) 4.70-4.83 (m, 1H) 5.51 (s, 2H) 7.53 (d, J=8.59 Hz, 1H) 7.77-7.87 (m, 2H)

**((1R,5S)-3-(5-chloro-2-((1-methyl-1H-pyrazol-4-yl)amino)pyrimidin-4-yl)-3,8-diazabicyclo[3.2.1]octan-8-yl)((S)-2,2-difluorocyclopropyl)methanone (Compound 29):** Synthesized in a similar manner to compound 7 in WO16027195 to provide 185 mg. MS m/z = 424.3 [M+H]^+^; HPLC RT = 2.41 min, purity = >99% [Waters Xbridge C18 50*2.0mm, 5um; 1% Acn (0.1%FA) in water (0.1%FA) to 5% over 0.6 min then 5% to 100% over 3.4 min flow rate 0.8 ml/min]; ^1^H NMR (400 MHZ, DMSO-d_6_) δ: 1.85-1.96 (m, 6H), 3.05-3.25 (m, 3 H), 3.80 (s, 3H), 4.14-4.18 (m, 2H), 4.61-4.68 (m, 2H), 7.46 (d, J=6.8Hz, 1 H), 7.73 (bs, 1H), 8.1 (d, J=5.6hz, 1H), 9.34 (bs, 1H)

**SI Table 2.** Phospho-STAT inhibition in lymphocyte assays IC_50_ of tofacitinib^13^ and PF-06673518 across wild type dog TYK2, cynomolgus monkey wild type TYK2. Values represent geomean IC50 (nM) ± standard deviation from four experiments.

| Compound | IL-12  (TYK2/JAK2)  induced pSTAT4 (nM) | | |
| --- | --- | --- | --- |
|  | Dog WT | Monkey WT |  |
| Tofacitinib | 265 ± 106 | 225 ± 68 |  |
| PF-06673518 | 679 ± 254 | 527 ± 147 |  |
